# Load-Bearing Performance of Non-Prismatic RC Beams Wrapped with Carbon FRP Composites

**DOI:** 10.3390/s23125409

**Published:** 2023-06-07

**Authors:** Suniti Suparp, Ali Ejaz, Kaffayatullah Khan, Qudeer Hussain, Panuwat Joyklad, Panumas Saingam

**Affiliations:** 1 Department of Civil and Environmental Engineering, Faculty of Engineering, Srinakharinwirot University, Nakhonnayok 26120, Thailand; suniti@g.swu.ac.th (S.S.); panuwatj@g.swu.ac.th (P.J.); 2 National Institute of Transportation, National University of Sciences and Technology (NUST), Islamabad 44000, Pakistan; enggaliejax@gmail.com; 3 Department of Civil and Environmental Engineering, College of Engineering, King Faisal University, Al-Hofuf 31982, Saudi Arabia; kkhan@kfu.edu.sa; 4 Dr. House Consultants Co., Ltd., Bangkok 10330, Thailand; ebbadat@hotmail.com; 5 Department of Civil Engineering, School of Engineering, King Mongkut’s Institute of Technology Ladkrabang, Bangkok 10520, Thailand

**Keywords:** non-prismatic, deflection, strain, CFRP strips, simply supported beams, shear, flexure

## Abstract

This study investigated the influence of CFRP composite wrapping techniques on the load–deflection and strain relationships of non-prismatic RC beams. A total of twelve non-prismatic beams with and without openings were tested in the present study. The length of the non-prismatic section was also varied to assess the effect on the behavior and load capacity of non-prismatic beams. The strengthening of beams was performed by using carbon fiber-reinforced polymer (CFRP) composites in the form of individual strips or full wraps. The linear variable differential transducers and strain gauges were installed at the steel bars to observe the load–deflection and strain responses of non-prismatic RC beams, respectively. The cracking behavior of unstrengthened beams was accompanied by excessive flexural and shear cracks. The influence of CFRP strips and full wraps was primarily observed in solid section beams without shear cracks, resulting in enhanced performance. In contrast, hollow section strengthened beams exhibited minor shear cracks alongside the primary flexural cracks within the constant moment region. The absence of shear cracks was reflected in the load–deflection curves of strengthened beams, which demonstrated a ductile behavior. The strengthened beams demonstrated 40% to 70% higher peak loads than control beams, whereas the ultimate deflection was increased up to 524.87% compared to that of the control beams. The improvement in the peak load was more prominent as the length of the non-prismatic section increased. A better improvement in ductility was achieved for the case of CFRP strips in the case of short non-prismatic lengths, whereas the efficiency of CFRP strips was reduced as the length of the non-prismatic section increased. Moreover, the load–strain capacity of CFRP-strengthened non-prismatic RC beams was higher than the control beams.

## 1. Introduction

Reinforced concrete (RC) structures are known to possess excessive self-weights. The issues related to the self-weights of RC structures become pronounced over long spans. Non-prismatic sections can significantly reduce the self-weights of RC structures. Additionally, non-prismatic sections impart aesthetically attractive architectural designs [1,2,3,4]. A desirable highlight in the construction of tall buildings is to reduce the ceiling heights that could be achieved using non-prismatic sections. These days, non-prismatic sections can be found in almost every kind of structure [5,6,7]. Openings inside beams can provide a safe passage for electrical, plumbing, and transmission works in addition to further lowering the self-weight of structures [8,9,10].

Despite having significantly lower weights and pleasing aesthetics, the structural performance of non-prismatic beams with hollow sections has not been investigated extensively. However, numerous studies exist investigating the behavior of non-prismatic and hollow sections separately. Vijayakumar and Madhavi [11] tested beams with circular openings concentrically placed along their neutral axes. The first cracking and ultimate loads of hollow section beams were found to be lower than their counterpart solid section beams. Abbass et al. [9] tested beams with square openings of 80 mm in size. The elastic modulus and ultimate load of the hollow section beam were lower than that of the solid section beam. El-kassas et al. [12] conducted experiments on nine deep beams with openings of different shapes, sizes, and locations. A reduction in load capacity was observed by the introduction of openings, whereas the magnitude of this reduction increased with the size of openings. The effect of the shape of openings on load capacity was found to be minimal. However, openings located within the compression zone had more impact on load capacity than the openings located in the tension zone. Abbass et al. [13] tested beams with square openings of side lengths 60 mm, 80 mm, and 100 mm. The ductility of beams with 60 mm and 80 mm openings was found to be higher than their corresponding solid section beams. However, the difference in ductility was negligible for a 100 mm opening. Elamary et al. [14] employed square openings inside RC beams with sizes ranging from 3% to 10% of the gross cross-sectional area. The results revealed that an opening with a core size lower than 10% of the gross area did not influence the load capacity significantly. However, the crack patterns were significantly influenced by the presence of openings, irrespective of their sizes. Balaji and Vetturayasudharsanan [7] examined the performance of hollow RC beams under two-point loading. The ultimate loads of hollow beams were found to be lower than that of the solid section beams. However, an opposite trend was observed in the case of ultimate deflections. 

There is a scarcity of experimental works that investigate the performance of non-prismatic beams. For this reason, the current ACI [15] and British [16] design codes lack specific guidance for the design of non-prismatic beams [17]. Tena-Colunga et al. [18] examined the performance of non-prismatic beams with and without shear reinforcement. The failure mechanism of non-prismatic beams was pronounced by the arching action within the haunched length that substantially differed from the behavior of prismatic beams. Caldentey et al. [19] found that a tapered section substantially reduced the ultimate capacity of beams in comparison to that of prismatic beams. Qissab and Salman [20] found a 45.5% reduction in the ultimate shear capacity when the tapered angle increased from 7° to 12°. 

The foregoing discussion concludes that a tapered or hollow section beam possesses lower structural performance than solid section beams. However, researchers have attempted to improve this behavior by different means. Hemzah et al. [8] introduced cast-in-place circular openings in RC beams. All beams with openings experienced higher deflections attributed to their lower stiffness as compared to the solid section beams, whereas a significant reduction in their ultimate capacities was observed. Another group of beams was wrapped using CFRP laminates to improve their performance. A significant improvement in the ultimate load and deflection characteristics was observed. Abbass et al. [9] introduced 0.5%, 1.0%, and 1.5% volume fractions of steel fibers to improve the flexural performance of hollow RC beams. The strengthened beams exhibited lower loads at first cracking and yielding. A substantial improvement in the peak load was observed, corresponding to the 1.5% volume fraction of steel fibers. Vijayakumar and Madhavi [11] introduced different volume fractions of steel fibers and a constant volume fraction of nylon fibers in the mix of hollow RC beams. A combination of 0.3% of steel fibers with 0.1% of nylon fibers resulted in the optimum improvement of the ultimate load capacity. 

It is noteworthy that the advantages associated with the use of FRP laminates for structural rehabilitation are manifested within their inherent characteristics. For instance, Mazucca et al. [21] found that glass fiber-reinforced polymers retained more than 50% of their tensile and compressive moduli values at room temperature when subjected to 200° Celsius. Xian et al. [22] investigated the effects of water immersion, bending loading, and the hybrid mode of carbon/glass fiber-reinforced polymer laminates. The pultrusion technique was used to develop sandwich structures of fiber laminates. The study revealed a reduction in flexural strength by approximately 15% and stiffness by around 12% in composite plates due to water immersion and freezing–thawing cycles. Bending strength decreased by approximately 18%, and bending stiffness declined by around 10% after exposure to freezing–thawing conditions. Alsuhaibani et al. [23] performed a study that involved the immersion of coupons from a common new CFRP laminate in heated water at 23, 45, and 60 °C for 224 days. After a period of 224 days of exposure, CFRP experienced a maximum decrease of 33% in its tensile capacity and 26% in its elastic modulus. Xian et al. [24] revealed that the prestressed CFRP plate, when exposed to hygrothermal and freeze–thaw environments, exhibited a decrease in mechanical performance over time. Specifically, the tensile capacity decreased by an average of 18%, and the elastic modulus decreased by approximately 12% during the exposure period. Despite possessing low resistance against high temperatures and natural weathering phenomena, FRP laminates have successfully been utilized in improving the structural behavior of reinforced concrete beams. Baggio et al. [25] enhanced the shear capacity of RC beams by using commercially manufactured carbon FRP (CFRP), glass FRP (GFRP), and fiber-reinforced cementitious matrix (FRCM) sheets. The FRP laminates were used with FRP anchors in some beams. The strengthened beams without anchors failed by the debonding of FRP laminates before diagonal shear failure, whereas the presence of anchors halted the debonding of FRP laminates. The experimental results of Ameli et al. [26] revealed that FRP wraps can increase the ultimate torque of fully wrapped beams substantially in addition to improving the ductility. Yang et al. investigated the strengthening of RC beams by using FRP laminates by incorporating corroded reinforcement. Applying the FRP-strengthening method directly to the beams without repairing the deteriorated concrete cover proved to be effective in enhancing both the load-carrying capacity and flexural stiffness of the beams.

It is of utmost importance to acknowledge that both the presence of openings and section tampering have damaging effects on the performance of concrete. Conversely, separate investigations focusing on the reinforcement of hollow or non-prismatic beams have revealed enhancements in their structural behavior. However, it is significant that, to the best of the author’s knowledge, no study has been conducted thus far to enhance the performance of hollow and non-prismatic beams through the application of carbon fiber-reinforced polymers (CFRP). The primary objective of this study is to address this research gap by conducting experiments on reinforced concrete (RC) beams, encompassing both beams with and without openings, as well as beams with prismatic and non-prismatic sections. By classifying the beams in this manner, it becomes feasible to compare the behavioral characteristics of prismatic and non-prismatic beams, as well as hollow and solid section beams and non-prismatic hollow and non-prismatic solid beams. Furthermore, this study aims to evaluate the effectiveness of CFRP in improving the performance of various types of beams, including hollow prismatic, hollow non-prismatic, solid prismatic, and solid non-prismatic beams. By thoroughly assessing the behavior of these different beam configurations, valuable insights can be gained regarding the potential benefits and limitations of CFRP in enhancing the structural performance of hollow and non-prismatic beams.

## 2. Experimental Program

### 2.1. Test Matrix

**Figure 1:**
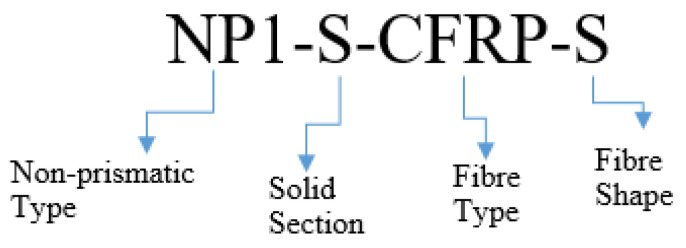
Identification scheme for beams.

The test matrix involved a total of twelve RC beams with non-prismatic sections. Two groups of beams were identified depending on the presence of openings. Group 1 beams comprised solid sections, whereas the beams in Group 2 were fabricated with inside openings. Each group contained two subgroups differentiated depending on the length of the non-prismatic section. Each subgroup comprised three beams: one beam was tested in an as-built condition and served as control, another beam with similar geometry was strengthened by using CFRP strips, and the last beam was strengthened by using full CFRP wraps. A four-part recognition was adopted to identify each beam. The first part identified the type of tapered angle, i.e., NP1 and NP2, for the non-prismatic length of type 1 and type 2, respectively. The second part identified the type of the section, i.e., H and S for the hollow section and solid section, respectively. The third part identified the presence of CFRP, i.e., CFRP and CON for CFRP-strengthened and control beams, respectively. The last part identified the type of CFRP strengthening, i.e., S and F for strips and full wraps, respectively. See Table 1 and Figure 1.

### 2.2. Details of Beams

**Figure 2:**
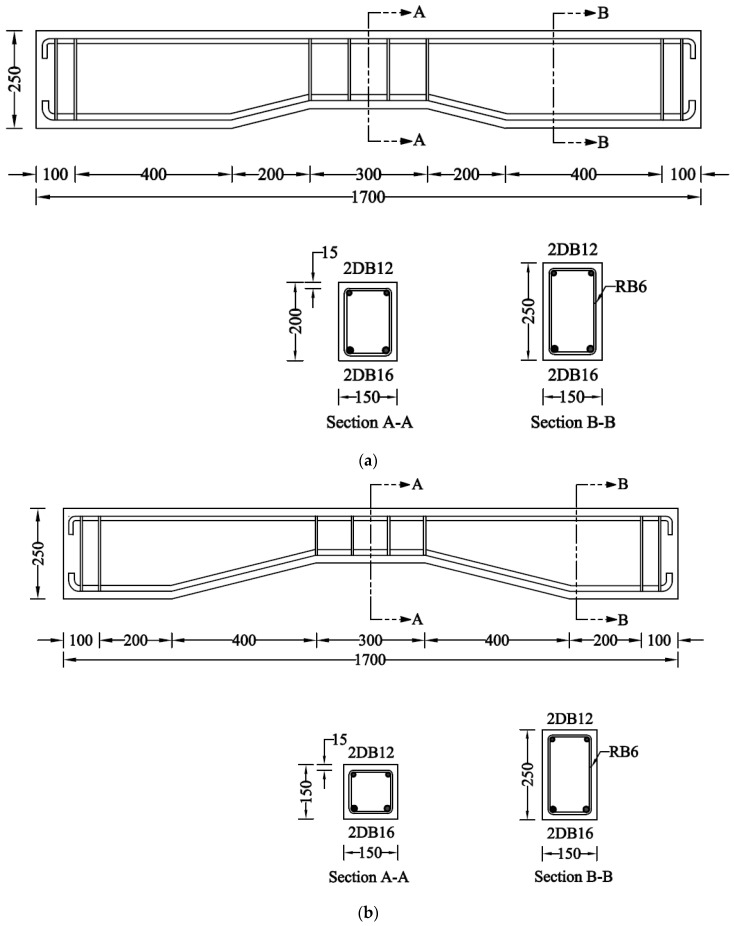

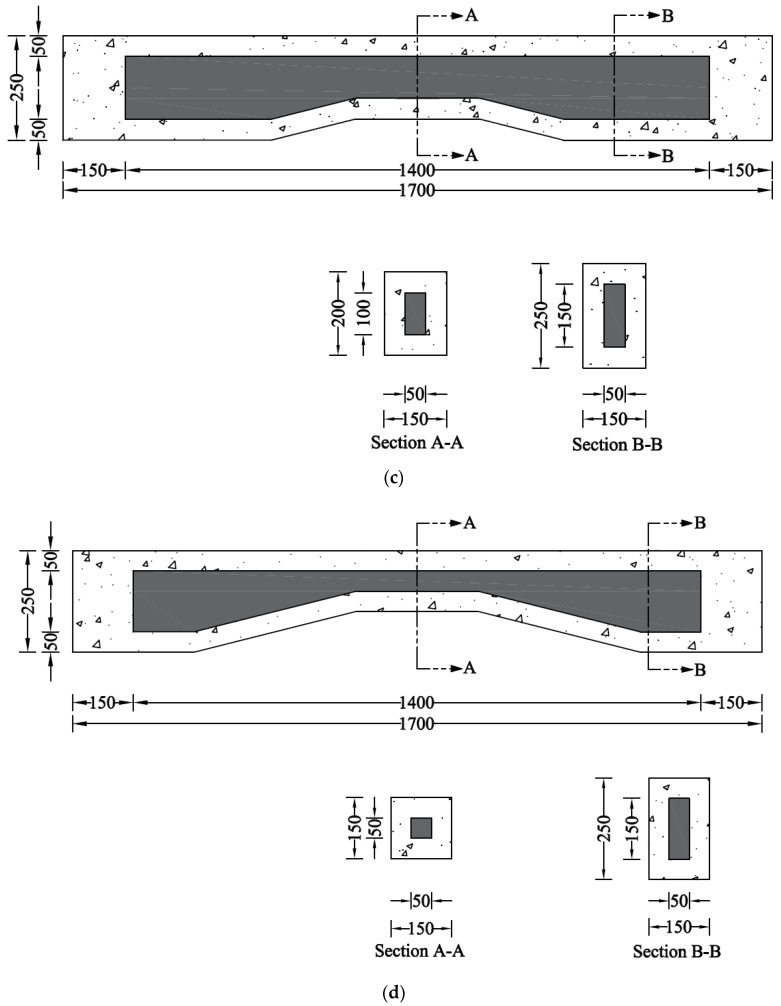
Details of control beams in each subgroup (units are in mm); (**a**) Beam NP1-S-CON; (**b**) Beam NP2-S-CON; (**c**) Beam NP1-H-CON; (**d**) Beam NP2-H-CON.

**Figure 3:**
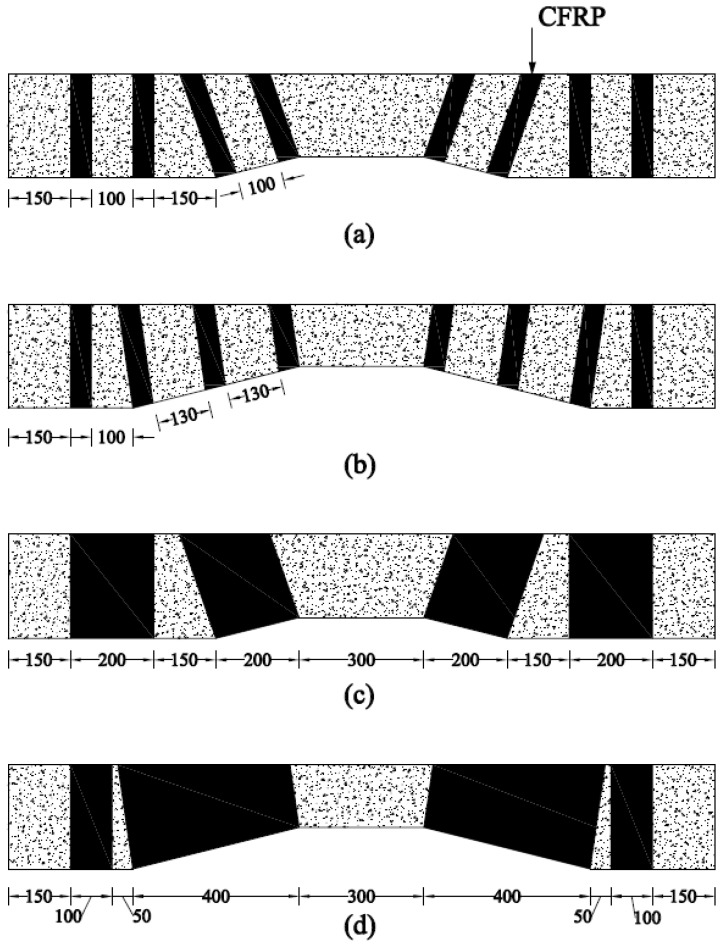
Details of CFRP-strengthened beams in each subgroup (**a**) NP1-S-CFRP-S, (**b**) NP2-S-CFRP-S, (**c**) NP1-S-CFRP-F, and (**d**) NP2-S-CFRP-F (units are in mm).

**Figure 4:**
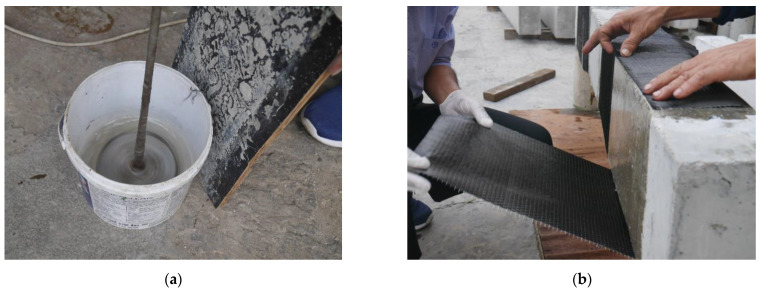

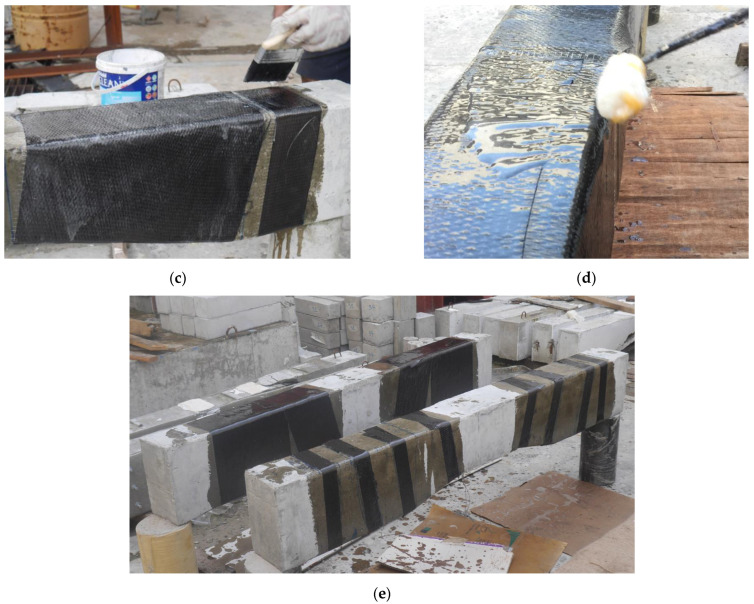
Typical strengthening process of beams; (**a**) Mixing of epoxy resin; (**b**) Application of carbon fiber; (**c**) Application of epoxy resin; (**d**) Use of roller; (**e**) Typical strengthened beams.

The details of the control beams are shown in Figure 2. The length of each beam was 1700 mm, with a width and height of 150 mm and 250 mm, respectively. Beams identified with NP1 had a prismatic length of 500 mm, measuring from each end, whereas beams identified with NP2 had a prismatic length of 300 mm measuring from each end. The hollow section beams comprised an opening of size 150 mm × 50 mm located at 150 mm from each end. The size of the opening was tapered in a geometrically similar manner to that of the gross cross-section. The opening size was reduced to 100 mm × 50 mm and 50 mm × 50 mm at midspans of beams type NP1 and NP2, respectively. Two types of strengthening schemes were employed, including CFRP strips and full CFRP wraps, as shown in Figure 3. The orientation of strips and wraps was synchronized with the section such that unidirectional fibers of CFRP remained perpendicular to the longitudinal axes of prismatic and non-prismatic sections. The strain gauges (gauge length = 5 mm) were obtained from Tokyo Measuring Instruments Lab and attached to the bottom steel bars i.e., DB16 (Figure 4a,b). It is important to note that the openings inside the beams were introduced by placing Styrofoam cut in the required dimensions, as shown in Figure 4c. The construction of beams was performed using plywood formwork as shown in Figure 4d. 

### 2.3. Material Properties

Each beam comprised two 12 mm-deformed bars as compression reinforcement, whereas two 16 mm-deformed bars were used as tension reinforcement. Both the top and bottom reinforcements were closed with 90-degree hooks. The prismatic section at midspan was reinforced against shear by 6 mm-round bars at 100 mm center-to-center spacing. The mechanical properties of steel bars were estimated by following the recommendations of ASTM E8/E8M-21 [27], as presented in Table 2. All beams were prepared by using ready-mix concrete. The compressive strength of concrete was estimated by testing standard concrete cylinders as per the recommendations of ASTM C39/C39M-21 [28]. In this study, carbon fiber and epoxy resin were obtained from Smart and Bright Co., Ltd., Bangkok, Thailand. The typical properties of the resin and carbon fiber sheets are provided in Table 3 and Table 4, respectively. 

### 2.4. Strengthening, Test Setup, and Instrumentation

**Figure 5:**
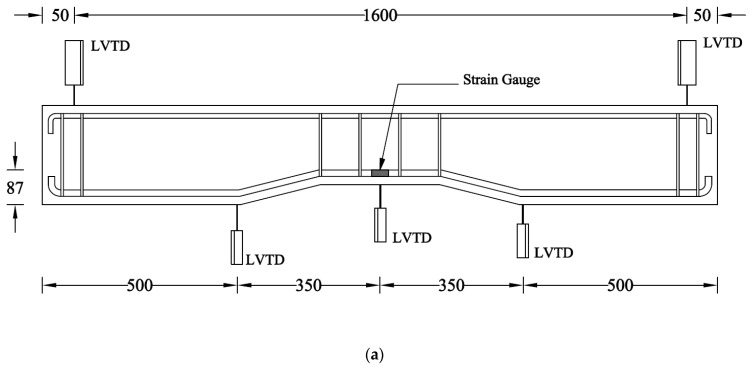

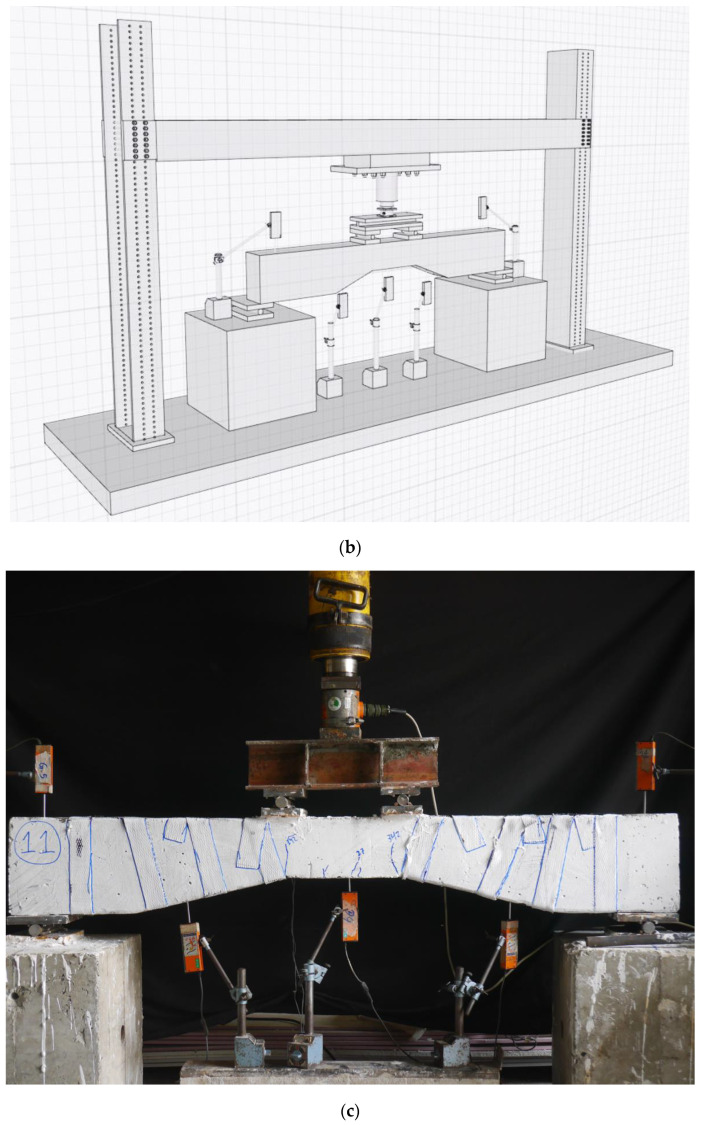
(**a**) Details of sensors in mm units, (**b**) schematic, and (**c**) actual test setup.

In this study, the strengthening of all beams was performed using carbon fiber-reinforced polymer composites. The strengthening was performed using a traditional hand layup method, as shown in Figure 4. A four-point bending setup was adopted, as shown in Figure 5. The load was applied using a hydraulic jack, whereas the point load was converted to two-point loading by using a steel spreader beam. A calibrated load cell (with a maximum capacity of 500 kN) was placed under the hydraulic jack to record the intensity of the applied load. Three displacement transducers recorded the vertical deflection of beams, whereas two additional displacement transducers recorded the uplift of supports, if any. The maximum measuring capacity of displacement transducers was 50 mm. The strain along the longitudinal bars was also monitored by attaching strain gages at the middle of the bottom longitudinal steel bars.

## 3. Experimental Results

### 3.1. Ultimate Failure Modes

**Figure 6:**
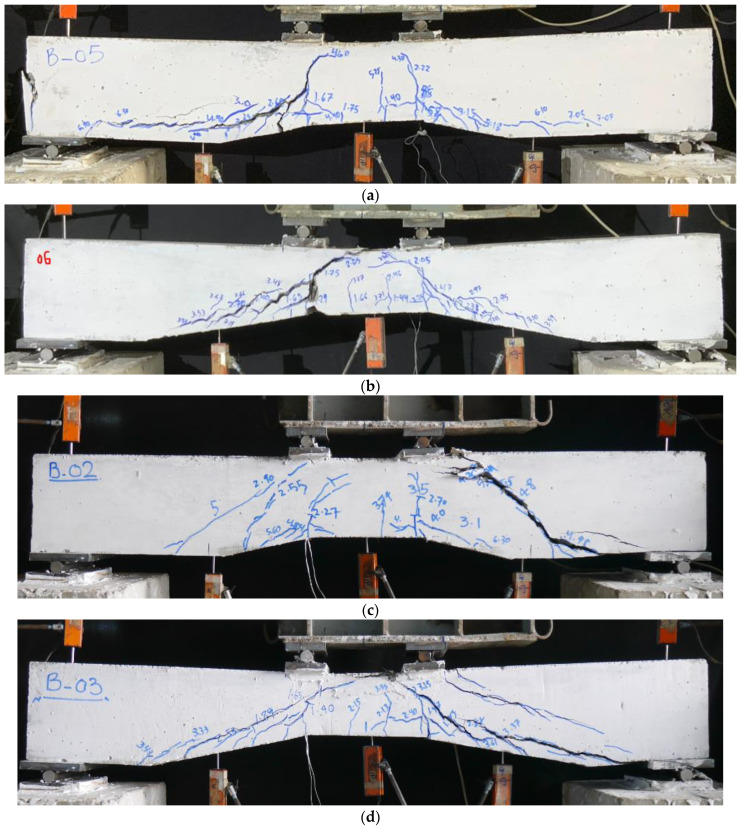
Ultimate failure modes of control beams; (**a**) NP1-S-CON; (**b**) NP2-S-CON; (**c**) NP1-H-CON; (**d**) NP2-H-CON.

The ultimate failure modes of the control beams are shown in Figure 6. Since a four-point bending test setup was adopted, it was expected that the maximum bending moment would be experienced within the constant moment region. The initial flexural cracks in all beams were observed within the constant moment region. As the loading increased, the number of flexural cracks increased. In addition, the existing vertical flexural cracks penetrated along the depth of beams in an inclined manner. The ultimate failure mode of Beam NP1-S-CON demonstrated numerous flexural cracks, whereas inclined cracks starting from the loading points followed the non-prismatic shape. These cracks characteristically represented the shear phenomenon and achieved a horizontal alignment within the prismatic length of shear spans. The length of the non-prismatic section in Beam NP2-S-CON was higher than that in Beam NP1-S-CON. The initiation of cracks at early loading stages in Beam NP2-S-CON was similar to that in Beam NP1-S-CON. However, a more extensive inclined cracking was observed in Beam NP2-1-CON. The hollow section control beams exhibited a similar cracking pattern as that exhibited by the solid section control beams. However, the number of main shear cracks was higher in hollow section control beams. In addition, the inclined length of shear cracks was extended to the supports, exhibiting a lower shear capacity in hollow section control beams. 

**Figure 7:**
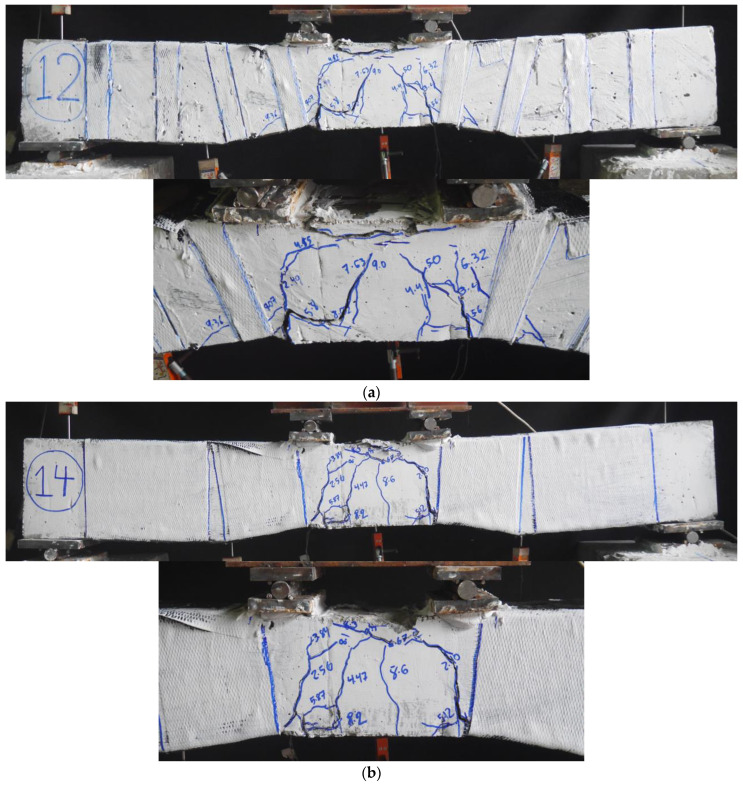

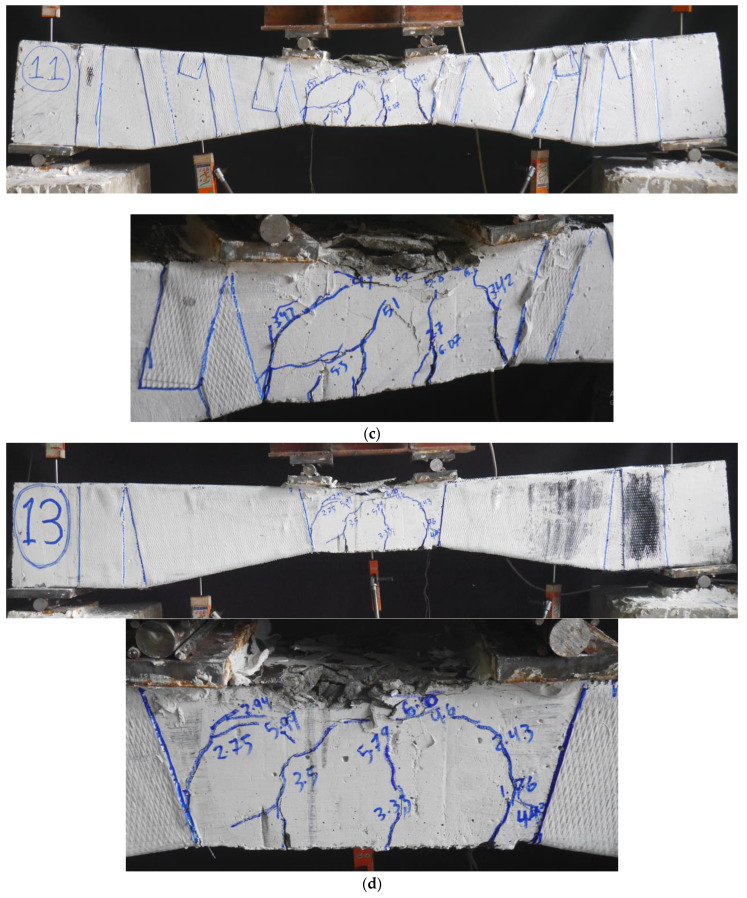

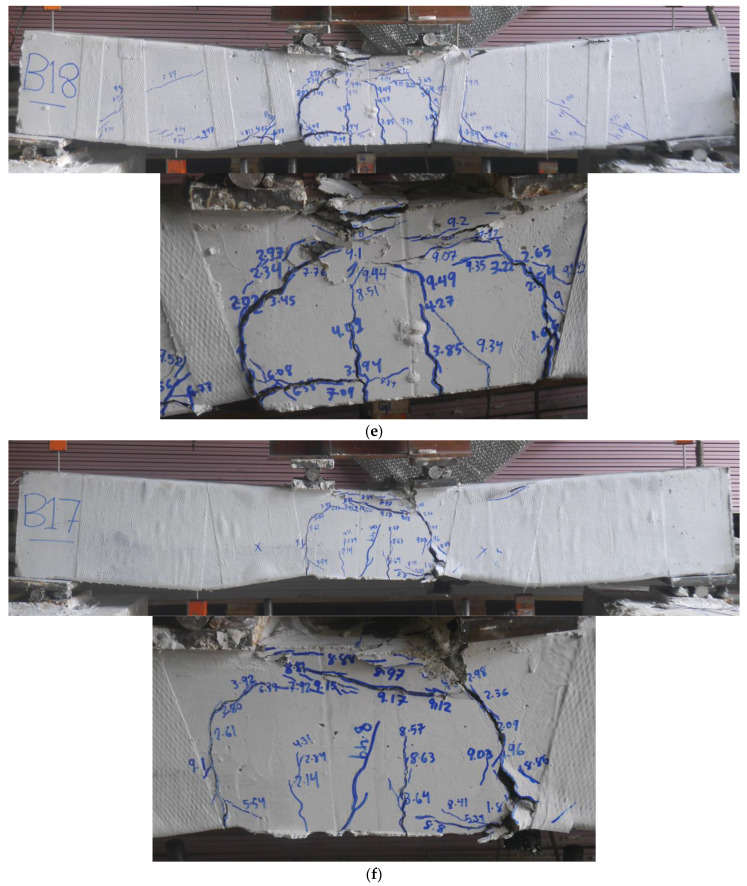

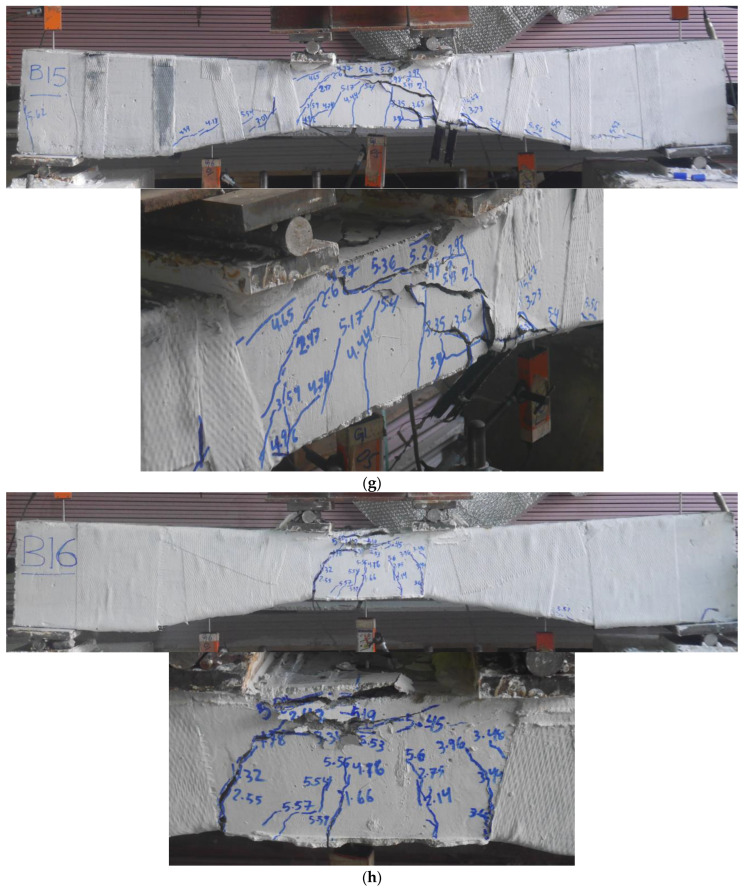
Ultimate failure modes of strengthened beams; (**a**) NP1-S-CFRP-S; (**b**) NP1-S-CFRP-F; (**c**) NP2-S-CFRP-S; (**d**) NP2-S-CFRP-F; (**e**) NP1-H-CFRP-S; (**f**) NP1-H-CFRP-F; (**g**) NP2-H-CFRP-S (**h**) NP2-H-CFRP-F.

**Figure 8:**
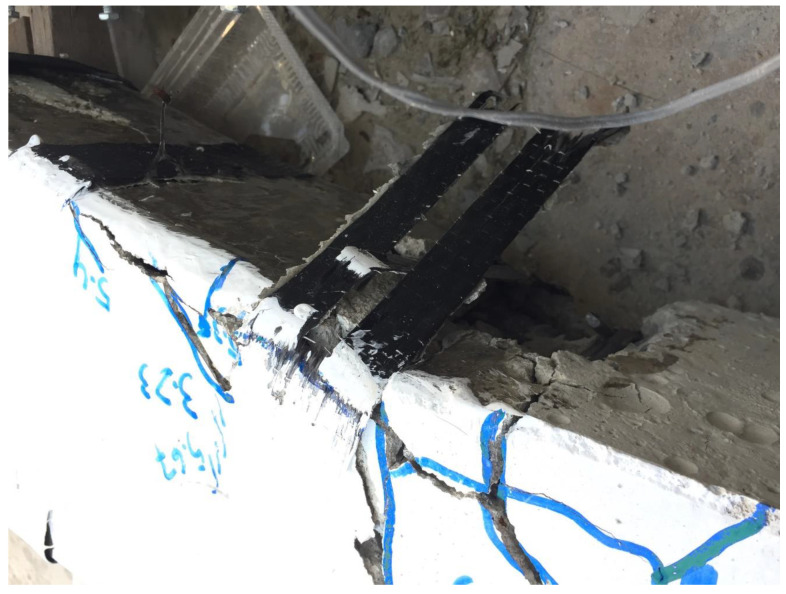
Delamination and rupture of CFRP.

The ultimate failure modes of strengthened beams are shown in Figure 7. All strengthened beams exhibited similar cracking at the initial loading stage, with all cracks being flexural and concentrated within the constant moment region. All strengthened beams exhibited extensive concrete crushing within the constant moment region. The impact of CFRP strips and full wraps were mainly observed in solid section beams where no shear cracks were observed, as shown in Figure 7. On the contrary, the hollow section strengthened beams demonstrated minor shear cracks in addition to the main flexural cracks within the constant moment region. For instance, Beam NP1-H-CFRP-S exhibited some shear cracks, as shown in Figure 7. It is important to note that no rupture of CFRP strips was observed. The delamination and rupture of CFRP strips were observed only in the case of NP1-S-CFRP-S, as shown in Figure 8.

The experimental findings revealed that control beams exhibited flexural cracks within the constant moment region, while strengthened solid section beams showed no shear cracks due to the effectiveness of CFRP strips and full wraps. However, the strengthened hollow section beams exhibited minor shear cracks alongside the main flexural cracks. The study provided valuable insights into the behavior and failure modes of non-prismatic beams, highlighting the importance of considering the interaction between flexural and shear stresses when designing and strengthening non-prismatic structures with CFRP reinforcement. Furthermore, the length of the non-prismatic zone needs significant attention when designing CFRP confinement. Figure 8 represents a failure mode of a strengthened beam, which includes the delamination of the CFRP composite. It is noteworthy that the debonding of CFRP undermines the full potential of CFRP composites and special care must be adopted to achieve a proper bond between CFRP and the concrete surface. 

### 3.2. Load–Displacement Curves

**Figure 9:**
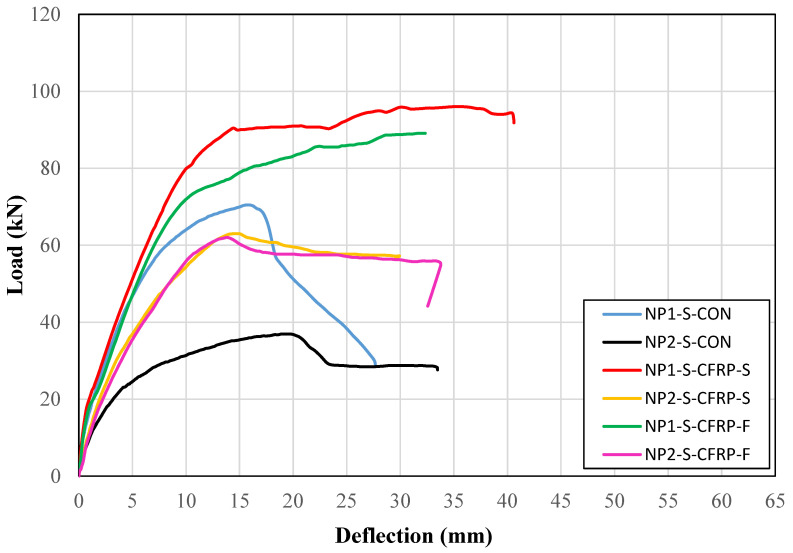
Load versus deflection curves of solid section beams.

The load–deflection curves of solid section beams are shown in Figure 9. It can be observed that the control Beam NP1-S-CON demonstrated a higher initial stiffness than the control Beam NP2-S-CON, which is attributed to the higher reduction in depth in Beam NP2-S-CON within the constant moment region. In addition, Beam NP1-S-CON was able to withstand a higher peak load than Beam NP2-S-CON. However, the ductility achieved by Beam NP2-S-CON was higher than that of Beam NP1-S-CON. The difference between the strip and full-wrap CFRP strengthening was prominent in beams with non-prismatic section type NP1. It is evident that Beam NP1-S-CFRP-S was able to achieve a higher peak load than Beam NP2-S-CFRP-F. However, their ductility and initial stiffness were inseparable. On the other hand, the improvement in the ductility, peak load, and initial stiffness of hollow section beams, imparted by strip and full-wrap CFRP strengthening, could not be distinguished. 

**Figure 10:**
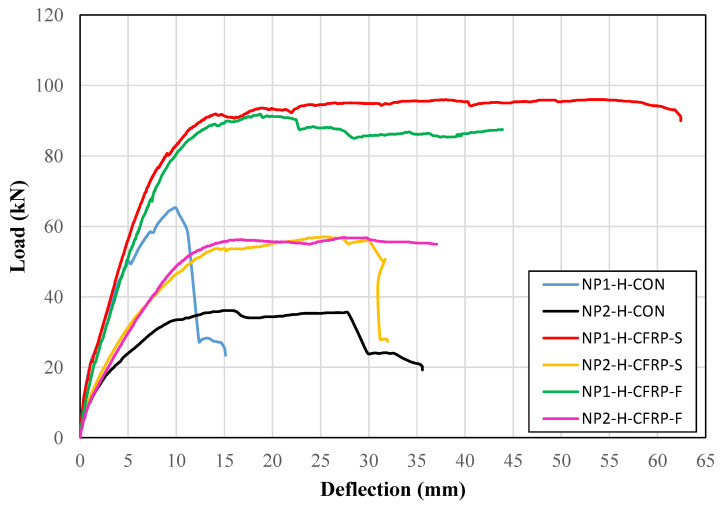
Load versus deflection curves of hollow section beams.

In a similar way, the load versus deflection curves of all hollow section beams are shown in Figure 10. A similar trend as that of solid section beams was observed. The control beam with the non-prismatic section of type NP1 was able to achieve a higher peak load than its counterpart beam with the non-prismatic section of type NP2. The strengthening by CFRP increased the initial stiffness in the case of the non-prismatic section of type NP2, whereas no such improvement was observed for the non-prismatic section of type NP1. For a longer non-prismatic length, there was no significant improvement in the ductility. However, substantial improvement in ductility was observed for beams with short non-prismatic lengths. 

The ongoing discussion suggests that the length of the non-prismatic section has a noticeable influence on the load–deflection behavior of the beams. The ductility and peak load-carrying capacity were significantly affected by the length of the non-prismatic section. In particular, a longer non-prismatic section had a detrimental effect on both the ductility and load-carrying capacity. Interestingly, the strip configuration of CFRP adopted in the present study was effective in enhancing the load-carrying capacity compared to that of the fully wrapped CFRP-strengthened beams. 

### 3.3. Peak Load and Deflection

The measured peak loads and ultimate deflections of all beams are presented in Table 5. Evidently, beams with the non-prismatic section of type NP2 experienced a greater improvement in peak load due to the CFRP strengthening. For instance, the increase in peak load for Beam NP1-S-CFRP-S was 36.26% greater than that of the control beam, whereas the same improvement in Beam NP2-S-CFRP-S was observed at 70.61%. This is because a longer non-prismatic length resulted in a higher reduction in cross-section in the case of beams with the NP2 section. As a result, the peak loads of control beams with the NP2 section were lower than that of the control beams with the NP1 section. Thus, it can be inferred that a greater improvement in the peak load can be expected as the length of the non-prismatic section, as well as the reduction in the overall cross-section, is increased. In the case of beams with the NP1 section, the impact of CFRP strips on ultimate deflection was found to be more prominent than that of CFRP wraps, whereas a greater improvement in the ultimate deflection of beams with the NP2 section was observed from the full wraps of CFRP. Thus, it can be inferred that a better improvement in ductility could be achieved for the case of CFRP strips when the length of the non-prismatic section is short, whereas the efficiency of CFRP strips is reduced as the length of the non-prismatic section is increased.

### 3.4. Energy Dissipation Capacity

The energy dissipation capacity of RC members is a desirable property, directly related to their ductile behavior. This was estimated by a summation of areas under load–deflection curves until the ultimate failure. It is to be noted that the ultimate failure point was taken to be the 20% drop in peak capacity in the case of softening behavior. Table 5 presents the energy dissipated by each beam and the percentage increase after strengthening by CFRP layers. It can be noted that a considerable increase in energy dissipation capacity was observed for all section and strengthening types. Particularly, hollow section beams with a Type-1 section demonstrated the maximum improvement in their energy dissipation capacity, i.e., up to 1121% improvement. It is vital to observe that the efficacy of CFRP confinement in improving energy dissipation capacity was subject to the length of the non-prismatic section. That is, a longer non-prismatic section demonstrated a lower increase in energy dissipation capacity as compared to the beams with a shorter non-prismatic section. Thus, it can be inferred that more confinement would be required to achieve the same ductility as that of a short non-prismatic length, as the length of the non-prismatic section is increased. 

### 3.5. Support Deflections

**Figure 11:**
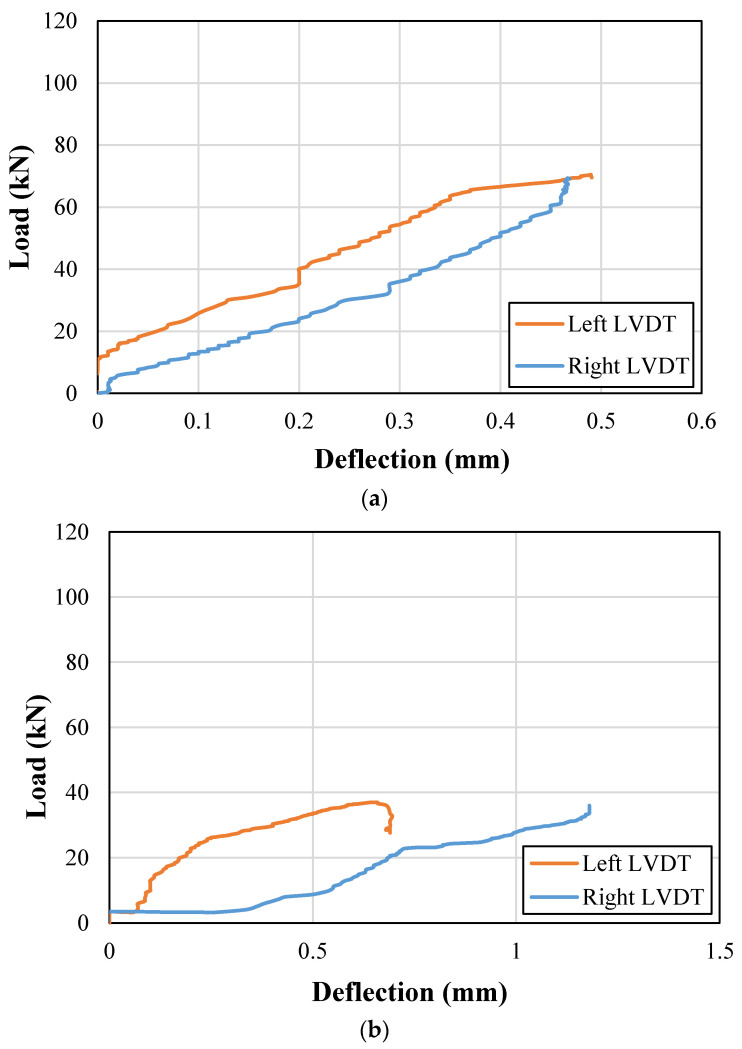

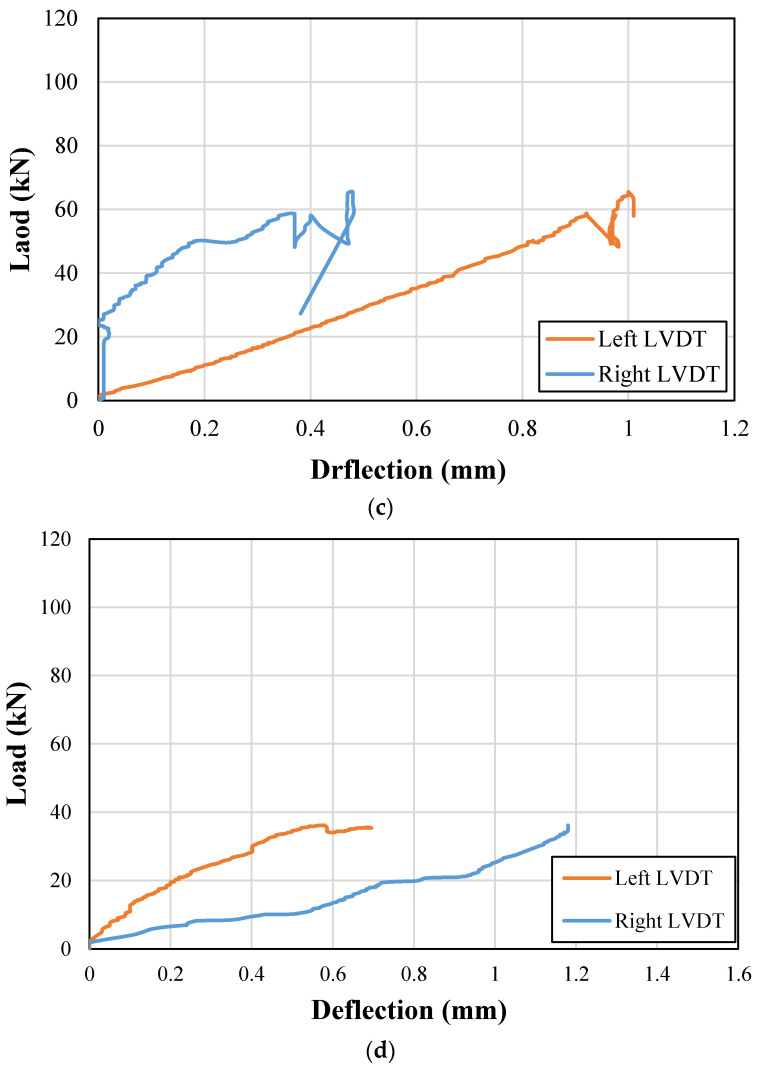
Support deflections for control beams; (**a**) NP1-S-CON; (**b**) NP2-S-CON; (**c**) NP1-H-CON; (**d**) NP2-H-CON.

**Figure 12:**
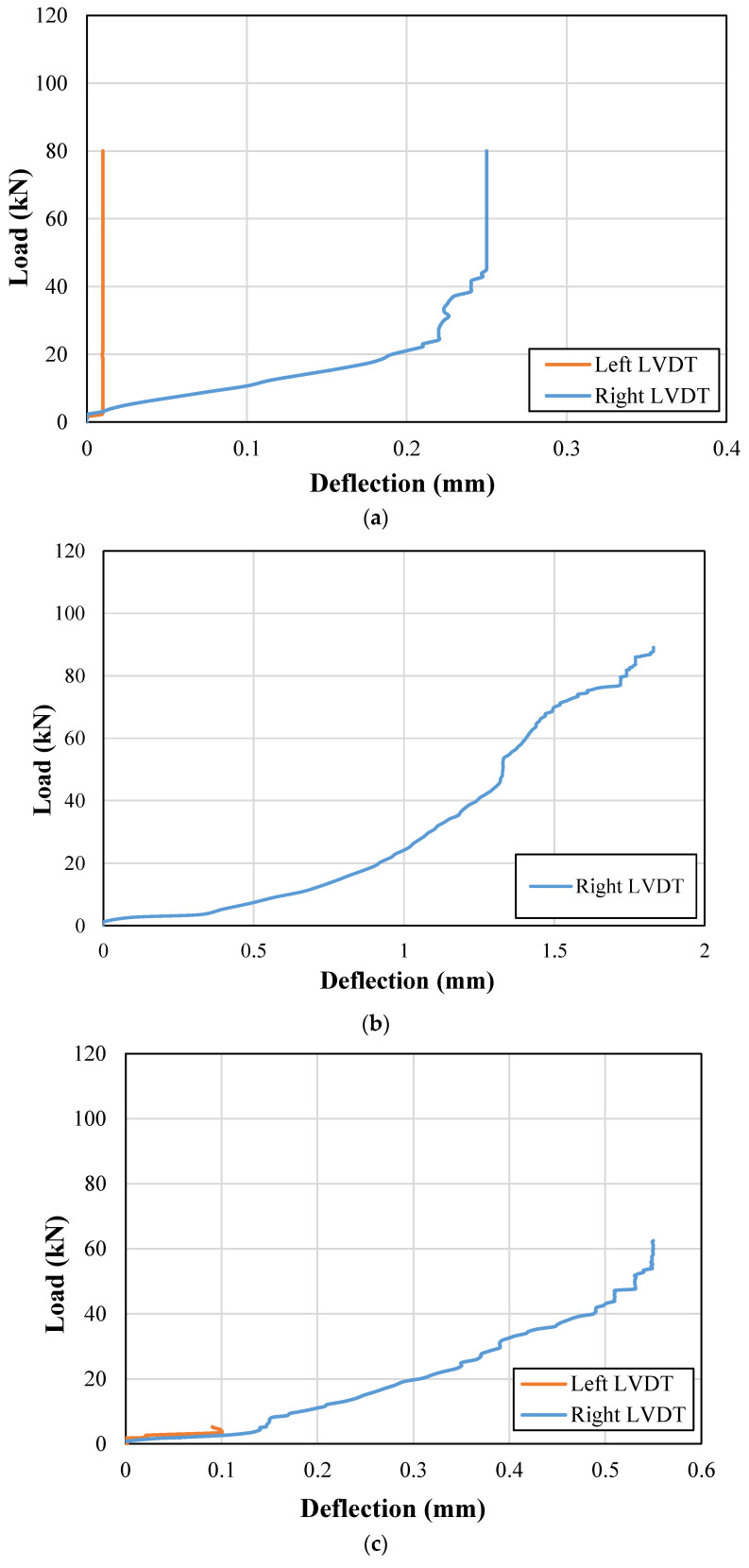

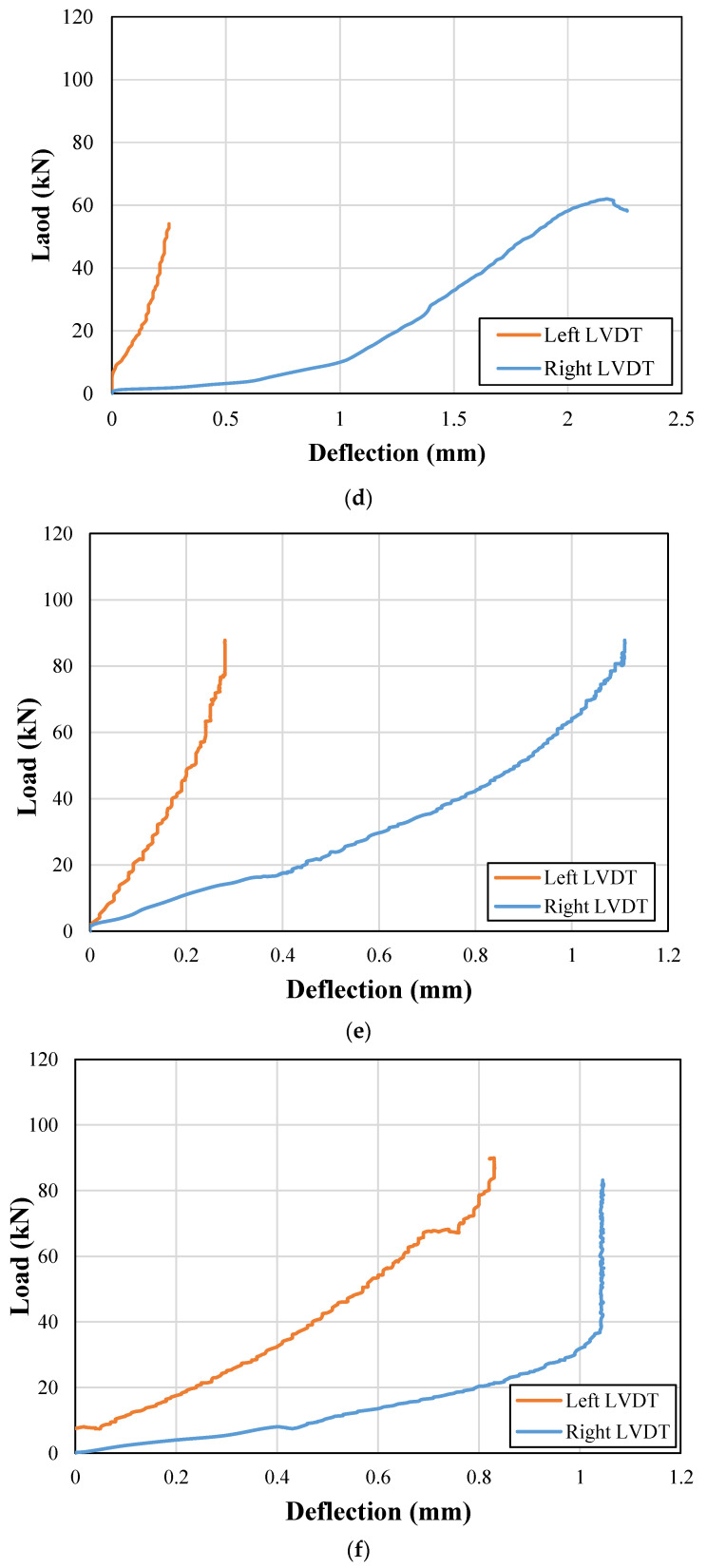

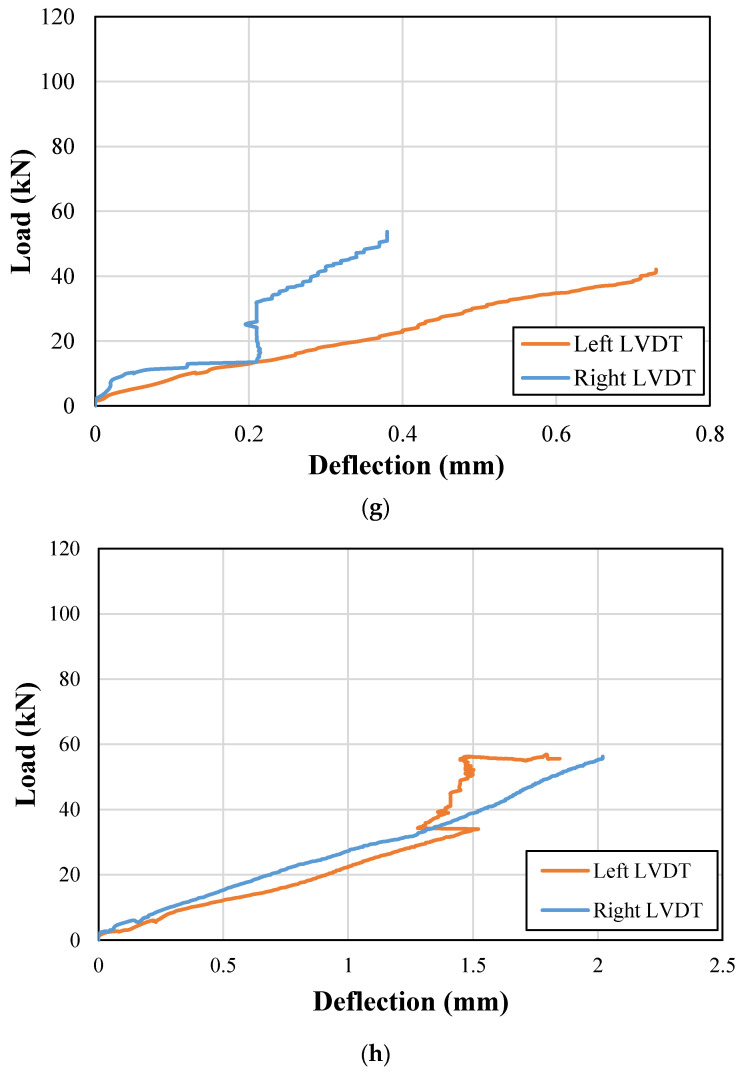
Support deflections for strengthened beams; (**a**) NP1-S-CFRP-S; (**b**) NP1-S-CFRP-F; (**c**) NP2-S-CFRP-S; (**d**) NP2-S-CFRP-F; (**e**) NP1-H-CFRP-S; (**f**) NP1-H-CFRP-F; (**g**) NP2-H-CFRP-S; (**h**) NP2-H-CFRP-F.

End deflections were also recorded during the load application on each beam. The recorded end deflection versus the applied load curves for control and strengthened beams are presented in Figure 11 and Figure 12, respectively. It can be observed that the support uplifts were mainly limited below the 3 mm mark in both the control and strengthened beams. Therefore, no correction for the support uplift was incorporated for the calculation of net midspan deflections. 

### 3.6. Strain along Longitudinal Bars

**Figure 13:**
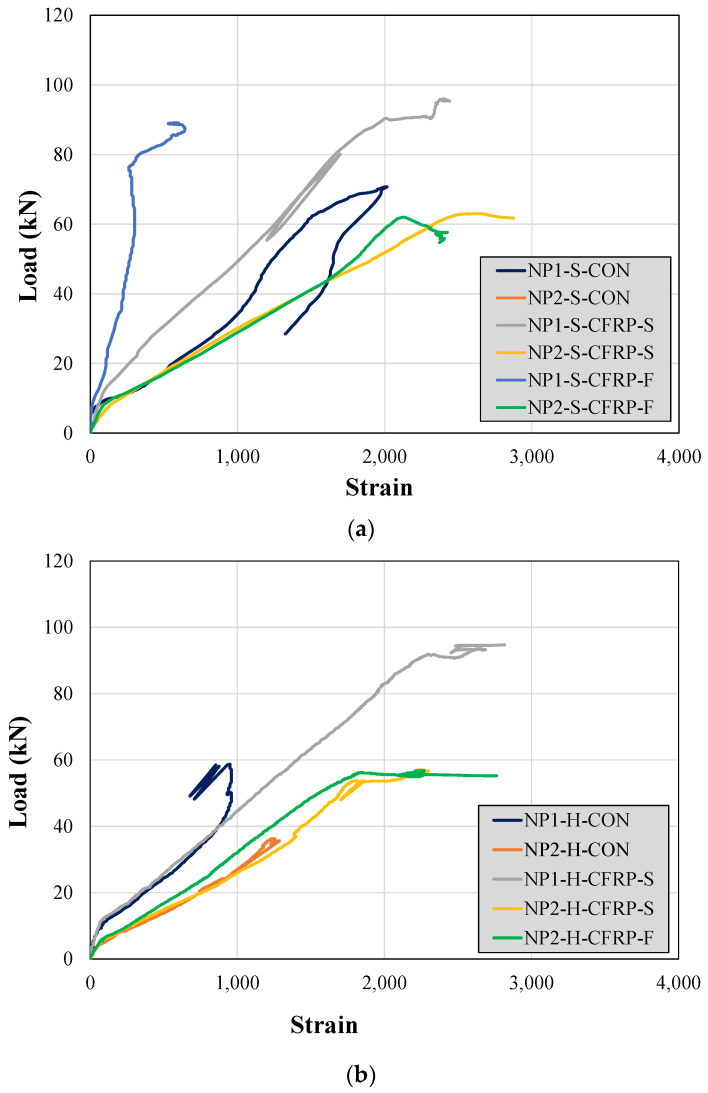
Steel strains along the bottom longitudinal bars in (**a**) solid section beams and (**b**) hollow section beams.

The recorded steel strains along the bottom longitudinal bars are shown in Figure 13. The effect of a longer non-prismatic section on the maximum longitudinal strain can be observed from the response beams NP1-S-CON and NP2-S-CON. The maximum strain experienced by the bottom steel bars in Beam NP1-S-CON was higher than that in Beam NP2-S-CON. However, this difference was marginal in the case of hollow section beams (i.e., see the response of beams NP1-H-CON and NP2-H-CON in Figure 13a). In addition, the longitudinal steel bars could not attain their yield limit in all the control beams. However, the strengthened beams exhibited the yielding of their bottom longitudinal bars. An exception was observed in the case of Beam NP1-S-CFRP-F, which can be attributed to the failure of the strain gage. 

**Figure 14:**
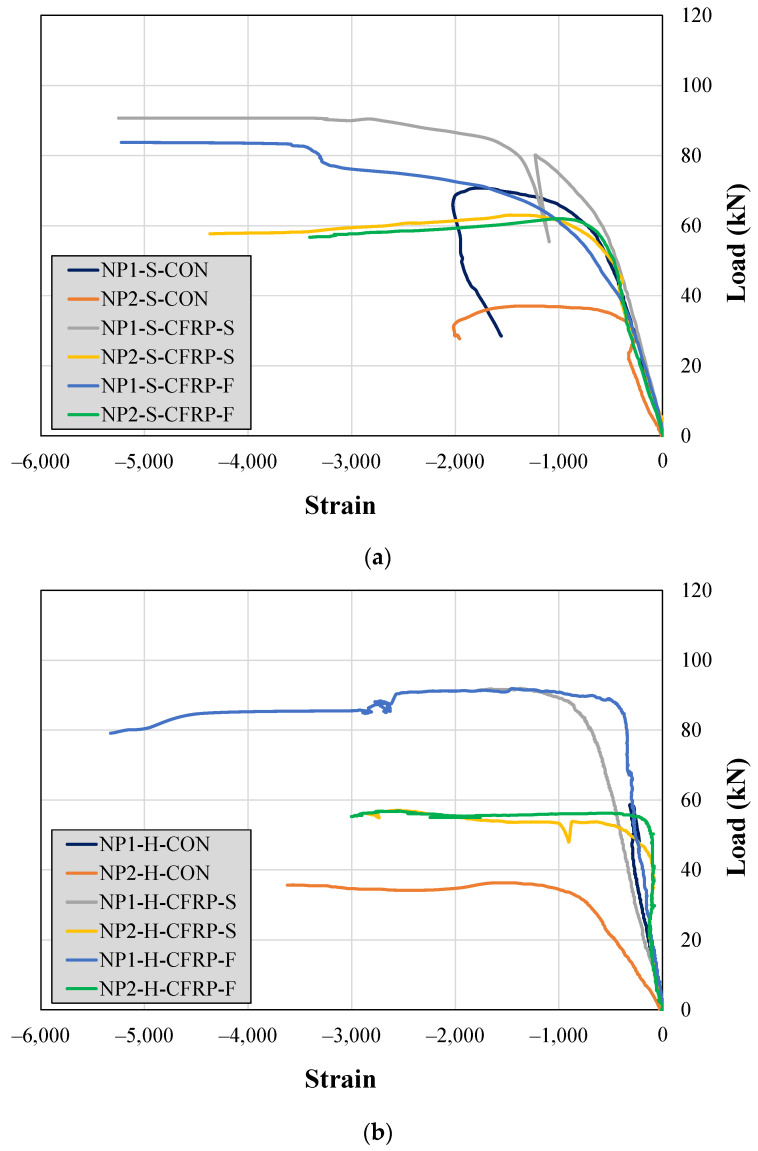
Steel strains along the top longitudinal bars in (**a**) solid section beams and (**b**) hollow section beams.

The strain along the top steel bars (i.e., the bars in compression) was also monitored during the experiments. The recorded strains are shown in Figure 14. Interestingly, the compression bars exhibited yielding in all the control beams except for Beam NP1-H-CON. However, the maximum negative strain was limited to 2000 microns in the control beams. In contrast, relatively higher negative strains were observed in CFRP-strengthened beams. 

## 4. Conclusions

This study presented experimental findings on non-prismatic, simply supported beams tested under four-point bending and strengthened with CFRP strips or full wraps. In addition, the length of the non-prismatic section was varied to assess its effect on the behavior and ultimate load capacity of the beams. The following conclusions were drawn from experimental findings.

1. The study findings showed that control beams developed flexural cracks within the constant moment region, whereas solid section beams strengthened with CFRP strips and full wraps exhibited no shear cracks. However, strengthened hollow section beams displayed minor shear cracks in addition to main flexural cracks. The research provided important insights into the behavior and failure modes of non-prismatic beams, emphasizing the significance of considering the interaction between flexural and shear stresses when designing and reinforcing such structures with CFRP.
2. It was also highlighted that the length of the non-prismatic section requires careful attention during CFRP confinement design. Some strengthened beams exhibited failure modes that involved CFRP delamination, emphasizing the need for a strong bond between CFRP and the concrete surface to fully utilize the potential of CFRP composites.
3. The load–deflection curves of control beams demonstrated an abrupt drop in their capacities soon after achieving the peak load, which can be attributed to the predominant shear phenomenon observed from the cracking behavior. On the contrary, the strengthened beams demonstrated ductile behavior.
4. The strengthened beams demonstrated 40% to 70% higher peak loads than control beams, whereas the ultimate deflection was increased up to 524.87% over that of the control beams. The improvement in the peak load was more prominent as the length of the non-prismatic section increased. A better improvement in ductility was achieved for the case of CFRP strips and short non-prismatic lengths, whereas the efficiency of CFRP strips was reduced as the length of the non-prismatic section increased.
5. Steel bars in tension did not achieve yielding in all control beams, whereas the strengthened beams exhibited yielding that was reflected in their ductile load–deflection response. It is noteworthy that both CFRP strips and full wraps allowed the beams to surpass yield points of longitudinal reinforcement in tension.

## Figures and Tables

**Table 1:** Test matrix.

Group	Subgroup	Type of Beam	CFRP Shape	Type of Prismatic Section	Section
1	1	NP1-S-CON	-	Type-01	Solid
		NP1-S-CFRP-S	Strip	Type-01	Solid
		NP1-S-CFRP-F	Full Wrap	Type-01	Solid
	2	NP2-S-CON	-	Type-02	Solid
		NP2-S-CFRP-S	Strip	Type-02	Solid
		NP2-S-CFRP-F	Full Wrap	Type-02	Solid
2	1	NP1-H-CON	-	Type-01	Hollow
		NP1-H-CFRP-S	Strip	Type-01	Hollow
		NP1-H-CFRP-F	Full Wrap	Type-01	Hollow
	2	NP2-H-CON	-	Type-02	Hollow
		NP2-H-CFRP-S	Strip	Type-02	Hollow
		NP2-H-CFRP-F	Full Wrap	Type-02	Hollow

**Table 2:** Mechanical properties of steel bars.

Type of Bar	Yield Strength (MPa)	Yield Strain (mm/mm)	Ultimate Strength (MPa)	Ultimate Strain (mm/mm)
RB-6	373	0.00184	494.7	0.1836
DB-12	633	0.00394	761	0.1151
DB-16	455	0.00241	614	0.0151

**Table 3:** Typical properties of resin.

Properties	Values	Units
Curing time	7–10	Hours
Compressive strength	650	kgf/cm^2^
Tensile strength	50	MPa
Elongation at break	2.5	%
Flexural strength	75	MPa

**Table 4:** Typical properties of carbon fiber sheets.

Properties	Values	Units
Weight	300	g/m^2^
Thickness	0.167	mm
Fiber density	1.8	g/cm^3^
Tensile strength	5214	MPa
Elongation	1.51	%

**Table 5:** Summary of peak loads and ultimate midspan deflections.

Beam	Peak Load (kN)	Increase in Peak Load (%)	Ultimate Deflection (mm)	Increase in Ultimate Deflection (%)	Dissipated Energy (kN-mm)	Increase in Dissipated Energy (%)
NP1-S-CON	70.47	-	15.63	-	955	-
NP1-S-CFRP-S	96.02	36.26	40.05	156.24	3313	246.50
NP1-S-CFRP-F	89.09	26.42	35.07	124.38	2300	140.84
NP2-S-CON	36.92	-	19.76	-	975	-
NP2-S-CFRP-S	62.99	70.61	30.00	51.82	1504	54.26
NP2-S-CFRP-F	62.02	67.98	34.11	72.62	1706	74.97
NP1-H-CON	65.25	-	9.97	-	447	-
NP1-H-CFRP-S	96.02	47.16	62.30	524.87	5462	1121.93
NP1-H-CFRP-F	91.85	40.77	43.90	340.32	3626	711.19
NP2-H-CON	36.25	-	22.50	-	820	-
NP2-H-CFRP-S	57.03	57.32	30.00	33.33	1131	37.93
NP2-H-CFRP-F	56.87	56.88	37.10	64.89	1752	113.66

## Data Availability

The data that support the findings of this study are available on request from the corresponding author.
